# Proteomic sample preparation for blast wound characterization

**DOI:** 10.1186/1477-5956-12-10

**Published:** 2014-02-14

**Authors:** Brett A Chromy, Angela Eldridge, Jonathan A Forsberg, Trevor S Brown, Benjamin C Kirkup, Eric Elster, Paul Luciw

**Affiliations:** 1 Department of Pathology and Laboratory Medicine, School of Medicine, University of California at Davis, Davis, CA, USA; 2 Center for Comparative Medicine, University of California at Davis, Davis, CA, USA; 3 Regenerative Medicine Department, Naval Medical Research Center, Silver Spring, MD, USA; 4 Department of Orthopaedics, National Military Medical Center, Bethesda, MD, USA; 5 Department of Wound Infections, Walter Reed Army Institute of Research, Silver Spring, MD, USA; 6 Department of Medicine, F Edward Hebert School of Medicine, Uniformed Services University of the Health Sciences, Bethesda, MD, USA; 7 Norman M. Rich Department of Surgery and Surgical Critical Care Institute, Uniformed Services University of the Health Sciences, Bethesda, MD, USA

**Keywords:** Blast wound, Proteomics, 2-D DIGE, Wound effluent, Biomarker discovery, Serum, High abundant protein removal

## Abstract

**Background:**

Blast wounds often involve diverse tissue types and require substantial time and treatment for appropriate healing. Some of these subsequent wounds become colonized with bacteria requiring a better understanding of how the host responds to these bacteria and what proteomic factors contribute wound healing outcome. In addition, using reliable and effective proteomic sample preparation procedures can lead to novel biomarkers for improved diagnosis and therapy.

**Results:**

To address this need, suitable sample preparation for 2-D DIGE proteomic characterization of wound effluent and serum samples from combat-wounded patients was investigated. Initial evaluation of crude effluent and serum proved the necessity of high abundant protein depletion. Subsequently, both samples were successfully depleted using Agilent Multiple Affinity Removal system and showed greatly improved 2-D spot maps, comprising 1,800 and 1,200 protein spots, respectively.

**Conclusion:**

High abundant protein removal was necessary for both wound effluent and serum. This is the first study to show a successful method for high abundant protein depletion from wound effluent which is compatible with downstream 2-D DIGE analysis. This development allows for improved biomarker discovery in wound effluent and serum samples.

## Introduction

Blast- and other combat wounds continue to be difficult to treat due to the complex interplay between the patient’s response to injury and the local wound environment [1-4]. A better understanding of the host systemic response to the injury and microbial colonization as well as the local wound microenvironment is essential in order to better identify a biomarker panel of predictors for wound healing or dehiscence [1]. Serum and effluent are both ideal biological samples for studying host proteins because they are representative of the current state of healing and the severity of microbial colonization. Serum is considered the most informative sample type for describing a patient’s current state of disease and systemic inflammatory response, because it contains a combination of all the differentiated sub-proteomes around the body [5]. Similarly, wound effluent is also considered a highly revealing biological fluid, because it directly reflects the wound site’s microenvironment which displays the damaged tissue’s current mechanisms of repair [6]. Because both sample types are easily obtained through minimally invasive procedures, they are ideal candidates for identification of biomarkers that can discriminate between stages of healing or microbial infection.

Techniques for biomarker discovery are constantly evolving to improve sensitivity and accuracy. Suitable preparation of the sample is critical for obtaining reliable and consistent results for proteomic analysis [7,8]. In addition, samples directly obtained from blood (serum) or containing portions of blood proteins (effluent) present significant analytical challenges for analyzing the full dynamic range of the complex proteome. The difficulties are derived from the vast concentration differences, from serum albumin ~45 mg/ml to lower abundant proteins as low as 1–10 pg/ml and all concentrations in between [5]. Current understanding is that biomarkers will originate from cellular interactions into blood and lymph or secretions from the affected tissue and will, therefore, be present in much lower concentrations than high abundant originating blood proteins [5]. Proteins with larger abundance mask those that are less abundant and need to be removed in order to analyze the lower abundant proteins to evaluate their usefulness as candidate biomarkers [9].

It is well established that serum gel-based proteomic analysis is greatly improved after removing high abundant proteins, but is the same true for wound effluent? Typically wound effluent consists of plasma, red and white blood cells, platelets, inflammatory proteins, enzymes and growth factors. Only a few research groups have performed 2-D-gel proteomics directly on wound effluent fluid and studied different types of wounds (chronic wound healing [6], leg ulcer wounds [10], snake venom damaged tissue [11]), each of which leads to a different combination of proteins comprising the fluid. However, effluent proteome of military combat blast wounds has yet to be analyzed or characterized. A consistently successful method for high abundant protein removal is Agilent’s Multiple Affinity Removal System [12-16], which is designed for blood plasma/serum and it has been successfully tested on other biological fluids, such as urine [16], CSF [17]. The Multiple Affinity Removal Column, nonetheless, has not been tested for effectiveness or compatibility on wound effluent. It is designed to specifically remove six high abundant proteins (albumin, IgA, IgG, antitrypsin, transferrin and haptoglobin) that comprise 85-90% of the total serum protein content, which results in an expected increase of loading capacity for lower abundant proteins by up to ten-fold [12]. Other methods for removing high abundant proteins exist including some that remove higher numbers of proteins, such as CaptureSelect (14 proteins) [18], IgY microbeads (12 proteins) [19], and MARS Hu-14 (14 proteins) and Proteoprep (20 proteins) [13]. However, we used the top-6 removal for wound effluent which provided a set of putative biomarkers for wound healing [20]. Several proteins found in that study would have been removed using these higher number abundant removal techniques.

In this study, we aim to (1) evaluate the proteome of crude wound effluent to determine whether effluent proteomic analysis would benefit by removal of high abundant proteins and (2) present a successful depletion method for serum and wound effluent that is compatible with improved downstream 2-D DIGE analysis.

## Materials and methods

### Sample collection

The study methodology is as reported elsewhere [1,3] and is reiterated here for completeness. In brief, serial effluent and serum samples were collected in an observational study with prospective data collection in accordance with the institutional review board of the Walter Reed National Military Medical Center (Bethesda, MD). All patients were evacuated to the National Capital Area from Iraq and Afghanistan that had sustained high-energy penetrating injuries to one or more extremities and were without confounding co-morbid conditions, such as immune disorders, connective tissue disorders, or any conditions requiring immunosuppressive agents, were eligible for inclusion. Surgical debridement, lavage, and negative-pressure wound therapy (NPWT) were repeated every 48–72 hours until surgical wound closure or coverage at the discretion of the attending surgeon and in accordance with current institutional standards of practice.

Wound effluent and serum samples were collected 2 hours following the first surgical debridement and over a 12-hour period prior to each subsequent wound debridement. Wound effluent samples (≥30 mL) were collected using the NPWT canister (without gel pack; Kinetic Concepts, Inc., San Antonio, TX). For serum samples, whole blood was collected in red top tubes and allowed to clot at room temperature for 30 min. Both samples were centrifuged at 2500 x g for 10 minutes to remove particulate matter and emboli. Effluent supernatants and serum were transferred to individually labeled polypropylene tubes, flash-frozen in liquid nitrogen, and stored at -80ºC until analysis.

### High abundant protein removal

Depletion of high abundant proteins was performed according to manufacturer’s instructions (Agilent Technologies). Briefly, human effluent or serum was diluted five times in Buffer A (40 μl sample and 160 μl of buffer, 200 μl total volume) and spun through a 0.22 micron spin filter tube (Millipore) at 16,000 x g for 5 min to remove particulates. Then effluent or serum was processed using 4.6 x 100 mm Multiple Affinity Removal Column Human-6 (Agilent Technologies), which specifically removes albumin, IgA, IgG, antitrypsin, transferrin and haptoglobin. A low abundant protein fraction was collected for each sample. Fractions were concentrated by precipitating with an equal volume of 20% trichloroacetic acid solution and incubated at 4°C for 30 min. Precipitate was spun down and washed twice with cold 100% acetone, allowed to air dry and then resuspended in DIGE labeling buffer (7 M urea, 2 M thiourea, 4% CHAPS, 30 mM Tris, pH 8.5). Protein quantification was performed using Precision Red Advanced Protein Assay Reagent (Cytoskeleton).

### SDS-PAGE

Crude and high abundant protein depleted effluent or serum samples (5 μg) were appropriately mixed with 5X sample loading buffer (0.2 M Tris pH 6.8, 20% glycerol, 10% SDS, 5% BME), boiled for 10 min at 100°C and resolved on a 4-20% Tris-Glycine gel (Invitrogen). The gel was stained for total protein using Sypro Ruby Protein Gel Stain (Invitrogen, S-12000) and visualized with UVP’s BioChemi system (UVP BioImaging Systems).

### 2-D DIGE

Crude and high abundant protein depleted effluent and serum samples were separated in 2 dimensions according to GE Life Sciences Ettan DIGE system protocol. Briefly, each sample (50 μg) was minimally labeled with 1 μl of 200 pM Cy2, Cy3 or Cy5 for 30 min. Labeling reactions were stopped by the addition of 1 μl of 1 mM lysine. The samples were pooled together and added to rehydration buffer (7 M urea, 2 M thiourea, 4% CHAPS, 1.2% DeStreak, 1% pharmalytes). A final volume of 450 μl sample was loaded onto 24 cm ph3-10NL Immobiline DryStrips (GE Life Sciences) and focused by active overnight rehydration, followed by isoelectric focusing for a total of 62,500 Vhrs. Strips were equilibrated in SDS equilibration buffer (6 M urea, 30% glycerol, 2% SDS) for 15 min with 10 mg/ml DTT, then 15 min in fresh buffer with 25 mg/ml 15 min with IAA, then applied to DIGE gels (GE Life Sciences) for 2nd dimension separation. The resulting CyDye labeled protein gels were scanned using 100 micron resolution on Typhoon 9410 (GE Life Sciences).

### Image analysis

Data analysis was carried out using DeCyder 2-D 7.0 software (GE Life Sciences). Spot detection and abundance quantification was performed using the differential in-gel analysis (DIA) module of DeCyder.

## Results

**Figure 1:**
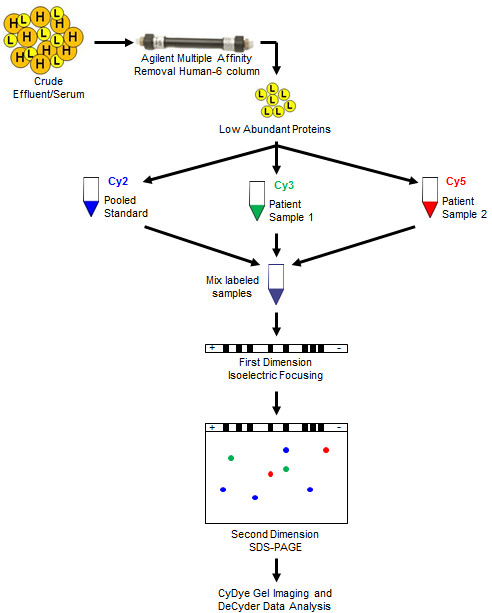
**Experimental design.** Crude effluent and serum were passed through a multiple affinity removal human top-6 column for high abundant protein removal. Low abundant proteins were used for Cy2 labeling and subsequent 2-D DIGE experimental analysis.

The key objective of this study was to evaluate the crude wound effluent proteome and identify a suitable sample preparation method for both wound effluent and serum for 2-D DIGE blast wound characterization, the approach is summarized in Figure 1. Crude serum is regularly subjected to high abundant protein removal prior to being analyzed by two dimensional electrophoresis [12,15,21,22], however there is little known about wound effluent proteomic sample preparation. Crude effluent and serum were independently subjected to high abundant protein removal using Agilent Human top-6 column according to manufacturer’s instructions. Removal efficacy was evaluated by SDS-PAGE and 2-D DIGE by comparing crude sample to after high abundant protein removal.

### Effluent and serum SDS-PAGE analysis after high abundant protein removal

**Figure 2:**
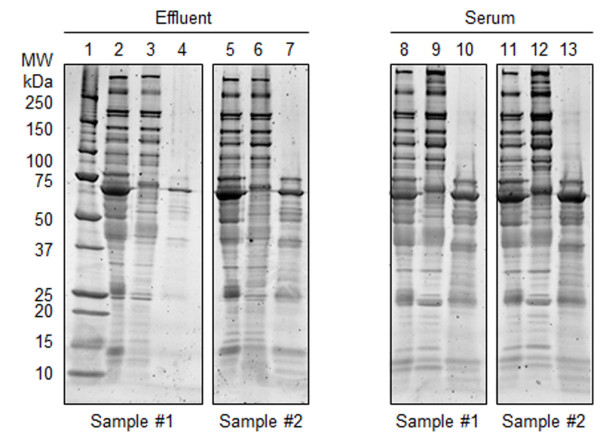
**SDS-PAGE of effluent and serum samples after high abundant protein removal.** Lane 1: Bio-Rad Precision Plus protein standard. Lanes 2 and 5: Crude effluent. Lanes 3 and 6: High abundant protein depleted effluent fraction. Lanes 4 and 7: Top-6 protein effluent fraction. Lanes 8 and 11: Crude serum. Lanes 9 and 12: High abundant protein depleted serum fraction. Lanes 10 and 13: Top-6 protein effluent fraction. Approximately 5 μg protein was loaded into each lane, proteins were visualized using SYPRO Ruby gel stain.

Initial evaluation of crude effluent (Figure 2, lanes 2 and 5) and serum (Figure 2, lanes 8 and 11) by SDS-PAGE displays effluent to have a very similar overall banding pattern when compared to serum. They both show the characteristic large, wide albumin band which clearly verifies the necessity of high abundant protein removal for both sample types. Corresponding low abundant fractions of effluent (Figure 2, lanes 3 and 6) and serum (Figure 2, lanes 9 and 12) show a much improved overall distribution of the proteomic bands. The high abundant protein fractions for effluent (Figure 2, lanes 4 and 7) and serum (Figure 2, lanes 10 and 13) illustrate removal of the targeted six high abundant proteins (albumin, IgG, IgA, transferrin, haptoglobin and antitrypsin). Additional detected bands in the high abundant protein fraction not positioned at one of the full kDa size proteins have been previously shown to be oligomers or fragments of one of these six proteins [15].

### 2-D DIGE analysis of effluent and serum after high abundant protein removal

**Figure 3:**
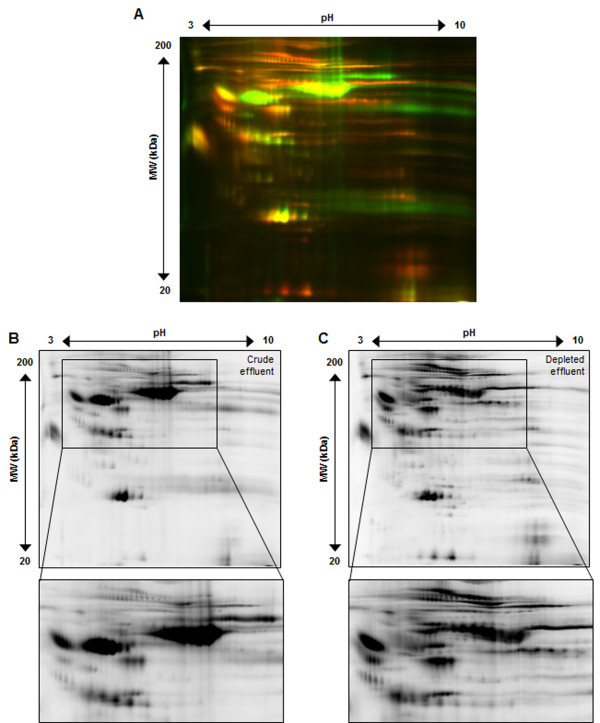
**High abundant protein removal improves spot number and resolution for wound effluent.** Panel **A** shows crude effluent (Cy3/green) and high abundant protein depleted effluent (Cy5/red) overlayed spot maps from the same initial patient sample analyzed by 2-D DIGE. Panel **B** and **C**, respectively, represent crude effluent (Cy3) and high abundant protein depleted effluent (Cy5). This illustrates the Agilent Multiple affinity removal top-6 human column worked for wound effluent and greatly improved the overall spot number and quality.

Crude effluent and high abundant depleted effluent (50 μg each) were individually labeled with Cy3 and Cy5, respectively, and separated in two dimensions, the resulting 2-D gel images are shown in Figure 3. Overlay (Figure 3A) of crude effluent (Cy3/green) and high abundant depleted effluent (Cy5/red) clearly demonstrates the specific removal of high abundant protein spots (primarily green spots), especially in the higher molecular weight section of the gel. High abundant depleted effluent enables the appearance of numerous lower abundant proteins (red spots) that are now detectable. Individual spot maps were analyzed by DeCyder resulting in 1000 protein spots detected in crude effluent (Figure 3B) and 1600 protein spots in high abundant depleted effluent (Figure 3C). Our data validates that crude wound effluent can be successfully depleted of high abundant proteins using Agilent’s Multiple Affinity Column (human top-6) and provide increased detection of lower abundant proteins by 2-D DIGE.

**Figure 4:**
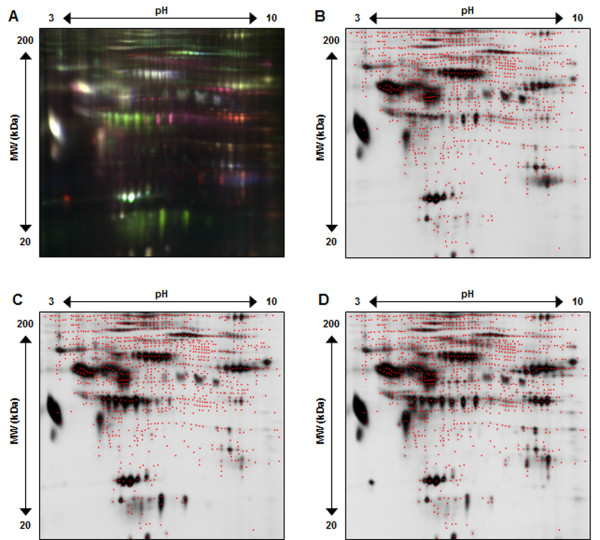
**Representative 2-D DIGE gel image of EFFLUENT after high abundant protein depletion.** Panel **A** shows all 3 CyDye fluorescent spot map images overlayed (Cy2/blue, Cy3/green, Cy5/red). Panel **B**, **C**, and **D** illustrate Cy2, Cy3 and Cy5 individual spot maps, where red dots define each of the 1800 total protein spots detected.

To evaluate the use of 2-D DIGE for detection of differential proteins in effluent after removal of high abundant proteins, three separate patient effluent samples were similarly depleted of high abundant proteins, labeled with Cy2, Cy3 and Cy5 and subsequently assessed by 2-D DIGE, the resulting gel images are shown in Figure 4. Figure 4A shows the overlay (Cy2/blue, Cy3/green, Cy5/red) of the three patient effluent samples and clearly displays the improved distribution of protein spots across the spot map, as well as the large variety in expression patterns for various proteins. Figure 4B-D presents the corresponding spot maps for each individual patient effluent sample. A maximum of 1800 protein spots were detected, red dots mark the center of each spot detected by DeCyder. When comparing to the 1000 total spots in crude effluent, high abundant protein removal significantly increases the overall number of detected protein spots thus increasing the number of potential proteins that can be assessed for biomarker discovery.

**Figure 5:**
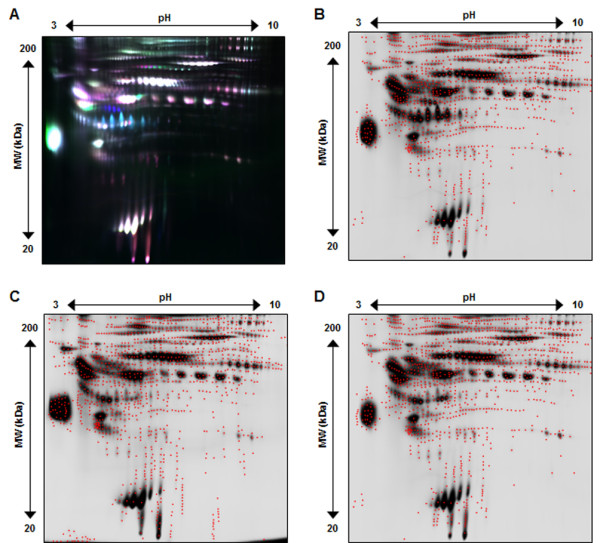
**Representative 2-D DIGE gel image of SERUM after high abundant protein removal.** Panel **A** shows all 3 CyDye fluorescent spot map images overlayed (Cy2/blue, Cy3/green, Cy5/red). Panel **B**, **C**, and **D** illustrate Cy2, Cy3 and Cy5 individual spot maps, where red dots define each of the 1200 total protein spots detected.

Similarly, serum after high abundant protein removal from three patients was evaluated by 2-D DIGE, the gel image is shown in Figure 5A-D. DeCyder analysis detected 1200 total protein spots, which is a similar to or exceeds other reports of greater than 1000 2-D gel spots [9,12,14,15,23,24].

## Discussion

A biomarker panel for wound effluent and serum from blast wound injured patients will help uncover the host mechanisms of systemic wound healing and response to microbial colonization. However, no single proteomic technique exists that can view all regions of the proteome simultaneously for these types of wide dynamic range biological fluids. Therefore, pre-fractionation or depletion is a sensible way for analyzing specific sections of the proteome that may reveal proteins of interest. This study focuses on (1) evaluating the necessity of high abundant protein removal for wound effluent and (2) developing an effective 2-D DIGE sample preparation method for investigating lower abundant proteins in wound effluent and serum.

Our data clearly demonstrates the similarity of wound effluent high/low abundant protein proportions to that of serum (Figures 2,3). In addition, we have successfully applied the Multiple Affinity Removal Column (human top-6) for depletion of wound effluent high abundant proteins which greatly improved the 2-D DIGE spot map (Figures 3,4). Our results indicate that this depletion method can be used for both wound effluent and serum reproducibly and reliably for the detection of differential proteins. Not only does this result in a dramatic increase in the visualization and resolution of lower abundant protein spots, but it also increases the probability of mass spectrometry identification of differential low abundant proteins of interest from 2-D DIGE gels.

Other techniques for high abundant protein removal exist and could be used to provide additional useful results for finding differential proteins. The decision to choose a particular affinity chromatography column will depend on what proteins might be problematic for the subsequent proteomic analysis and whether potential proteins of interest would be removed. Columns that remove more than the top-6 proteins are able to deplete additional abundant proteins, which could increase the number of low abundance proteins for subsequent proteomics analysis. For example, one study used three removal techniques on human plasma and found that all three gave complementary and overlapping, but different results [13]. Since this report is the first to describe the high abundant protein removal from wound effluent, it would be of value to include different removal techniques in subsequent studies to complement the results found here. Another important determination for which high abundant protein removal technique to use involves the decision to use affinity column chromatography or affinity bead capture. Each technique has benefits, but chromatography provides exceptionally low sample to sample variability [25], specifically by relying on the use of a chromatography system that can reduce potential sample preparation errors. Not only should additional affinity depletion techniques be used to study wound effluent, but other types of high abundant protein removal, such as the use of hydrogel particles [26] should provide benefits forwards biomarker discovery.

Our data provides the necessary method development to study the host proteome response to blast wound injury both systemically (serum) and locally (effluent) using the 2-D DIGE platform. This approach should allow for the detection and development of novel biomarker panels beyond those that are currently commercially available which offers the potential to improve clinical care.

## Competing interests

The authors declare that they have no competing interests.

## Authors’ contribution

BAC, AE, and PAL initially conceived of and designed the proteomics experiments. The overall design of the study, including planning and various aspects of interpretation of results, involved BAC, AE, JF, TSB, BK, EE and PAL. AE and BAC acquired the data. TSB and EE supplied crucial samples and clinical information. AE PL and BAC generated the initial draft of the manuscript. All authors read and approved the final manuscript.

## Disclaimer

The views expressed in this manuscript are those of the authors and do not reflect the official policy of the Department of the Army, Department of the Navy, the Department of Defense or the United States Government.

This effort was supported (in part) by the U.S. Navy Bureau of Medicine and Surgery under the Medical Development Program and Office of Naval Research.

Several co-authors are military service members (or employee of the U.S. Government). This work was prepared as part of their official duties. Title 17 U.S.C. 105 provides the “Copyright protection under this title is not available for any work of the United States Government.” Title 17 U.S.C. 101 defines a U.S. Government work as a work prepared by a military service member or employee of the U.S. Government as part of that person’s official duties.

I/We certify that all individuals who qualify as authors have been listed; each has participated in the conception and design of this work, the analysis of data (when applicable), the writing of the document, and the approval of the submission of this version; that the document represents valid work; that if we used information derived from another source, we obtained all necessary approvals to use it and made appropriate acknowledgements in the document; and that each takes public responsibility for it.

## References

[B1] Brown TS Hawksworth JS Sheppard FR Tadaki DK Elster E Inflammatory response is associated with critical colonization in combat wounds Surg Infect (Larchmt) 2011 12 5 351 357 10.1089/sur.2010.110 21936666

[B2] Forsberg JA Elster EA Andersen RC Nylen E Brown TS Rose MW Stojadinovic A Becker KL McGuigan FX Correlation of procalcitonin and cytokine expression with dehiscence of wartime extremity wounds J Bone Joint Surg Am 2008 90 3 580 588 10.2106/JBJS.G.00265 18310708

[B3] Hawksworth JS Stojadinovic A Gage FA Tadaki DK Perdue PW Forsberg J Davis TA Dunne JR Denobile JW Brown TS Elster EA Inflammatory biomarkers in combat wound healing Ann Surg 2009 250 6 1002 1007 10.1097/SLA.0b013e3181b248d9 19953718

[B4] Evans KN Forsberg JA Potter BK Hawksworth JS Brown TS Andersen R Dunne JR Tadaki D Elster EA Inflammatory cytokine and chemokine expression is associated with heterotopic ossification in high-energy penetrating war injuries J Orthop Trauma 2012 26 11 e204 e213 10.1097/BOT.0b013e31825d60a5 22588530

[B5] Jacobs JM Adkins JN Qian WJ Liu T Shen Y Camp DG II Smith RD Utilizing human blood plasma for proteomic biomarker discovery J Proteome Res 2005 4 4 1073 1085 10.1021/pr0500657 16083256

[B6] Eming SA Koch M Krieger A Brachvogel B Kreft S Bruckner-Tuderman L Krieg T Shannon JD Fox JW Differential proteomic analysis distinguishes tissue repair biomarker signatures in wound exudates obtained from normal healing and chronic wounds J Proteome Res 2010 9 9 4758 4766 10.1021/pr100456d 20666496

[B7] Fountoulakis M Proteomics: current technologies and applications in neurological disorders and toxicology Amino Acids 2001 21 4 363 381 10.1007/s007260170002 11858696

[B8] Fountoulakis M Takacs B Enrichment and proteomic analysis of low-abundance bacterial proteins Methods Enzymol 2002 358 288 306 12474394 10.1016/s0076-6879(02)58096-4

[B9] Fernandez-Costa C Calamia V Fernandez-Puente P Capelo-Martinez JL Ruiz-Romero C Blanco FJ Sequential depletion of human serum for the search of osteoarthritis biomarkers Proteome Sci 2012 10 1 55 10.1186/1477-5956-10-55 22971006 PMC3515479

[B10] Fernandez ML Broadbent JA Shooter GK Malda J Upton Z Development of an enhanced proteomic method to detect prognostic and diagnostic markers of healing in chronic wound fluid Br J Dermatol 2008 158 2 281 290 18070206 10.1111/j.1365-2133.2007.08362.x

[B11] Escalante T Rucavado A Pinto AF Terra RM Gutierrez JM Fox JW Wound exudate as a proteomic window to reveal different mechanisms of tissue damage by snake venom toxins J Proteome Res 2009 8 11 5120 5131 10.1021/pr900489m 19764775

[B12] Bjorhall K Miliotis T Davidsson P Comparison of different depletion strategies for improved resolution in proteomic analysis of human serum samples Proteomics 2005 5 1 307 317 10.1002/pmic.200400900 15619298

[B13] Yadav AK Bhardwaj G Basak T Kumar D Ahmad S Priyadarshini R Singh AK Dash D Sengupta S A systematic analysis of eluted fraction of plasma post immunoaffinity depletion: implications in biomarker discovery PLoS One 2011 6 9 e24442 10.1371/journal.pone.0024442 21931718 PMC3168506

[B14] Polaskova V Kapur A Khan A Molloy MP Baker MS High-abundance protein depletion: comparison of methods for human plasma biomarker discovery Electrophoresis 2010 31 3 471 482 10.1002/elps.200900286 20119956

[B15] Chromy BA Gonzales AD Perkins J Choi MW Corzett MH Chang BC Corzett CH McCutchen-Maloney SL Proteomic analysis of human serum by two-dimensional differential gel electrophoresis after depletion of high-abundant proteins J Proteome Res 2004 3 6 1120 1127 10.1021/pr049921p 15595720

[B16] Kushnir MM Mrozinski P Rockwood AL Crockett DK A depletion strategy for improved detection of human proteins from urine J Biomol Tech 2009 20 2 101 108 19503621 PMC2685607

[B17] Ogata Y Charlesworth MC Muddiman DC Evaluation of protein depletion methods for the analysis of total-, phospho- and glycoproteins in lumbar cerebrospinal fluid J Proteome Res 2005 4 3 837 845 10.1021/pr049750o 15952730

[B18] Kullolli M Warren J Arampatzidou M Pitteri SJ Performance evaluation of affinity ligands for depletion of abundant plasma proteins J Chromatogr B Analyt Technol Biomed Life Sci 2013 939 10 16 24090752 10.1016/j.jchromb.2013.09.008

[B19] Huang L Harvie G Feitelson JS Gramatikoff K Herold DA Allen DL Amunngama R Hagler RA Pisano MR Zhang WW Fang X Immunoaffinity separation of plasma proteins by IgY microbeads: meeting the needs of proteomic sample preparation and analysis Proteomics 2005 5 13 3314 3328 10.1002/pmic.200401277 16041669

[B20] Chromy BA Eldridge A Forsberg JA Brown TS Kirkup BC Jaing C Be NA Elster E Luciw PA Wound outcome in combat injuries is associated with a unique set of protein biomarkers J Transl Med 2013 11 1 281 10.1186/1479-5876-11-281 24192341 PMC3827499

[B21] Bellei E Bergamini S Monari E Fantoni LI Cuoghi A Ozben T Tomasi A High-abundance proteins depletion for serum proteomic analysis: concomitant removal of non-targeted proteins Amino Acids 2011 40 1 145 156 10.1007/s00726-010-0628-x 20495836

[B22] Stempfer R Kubicek M Lang IM Christa N Gerner C Quantitative assessment of human serum high-abundance protein depletion Electrophoresis 2008 29 21 4316 4323 10.1002/elps.200800211 18956433

[B23] Corzett TH Fodor IK Choi MW Walsworth VL Turteltaub KW McCutchen-Maloney SL Chromy BA Statistical analysis of variation in the human plasma proteome J Biomed Biotechnol 2010 2010 258494 20130815 10.1155/2010/258494 PMC2814230

[B24] Yu KH Rustgi AK Blair IA Characterization of proteins in human pancreatic cancer serum using differential gel electrophoresis and tandem mass spectrometry J Proteome Res 2005 4 5 1742 1751 10.1021/pr050174l 16212428

[B25] Corzett TH Fodor IK Choi MW Walsworth VL Chromy BA Turteltaub KW McCutchen-Maloney SL Statistical analysis of the experimental variation in the proteomic characterization of human plasma by two-dimensional difference gel electrophoresis J Proteome Res 2006 5 10 2611 2619 10.1021/pr060100p 17022632

[B26] Such-Sanmartin G Ventura-Espejo E Jensen ON Depletion of abundant plasma proteins by Poly(N-isopropylacrylamide-acrylic acid) Hydrogel particles Anal Chem 2014 86 3 1543 1550 10.1021/ac403749j 24428553

